# Early warning model for passenger disturbance due to flight delays

**DOI:** 10.1371/journal.pone.0239141

**Published:** 2020-09-21

**Authors:** Yunyan Gu, Jianhua Yang, Conghui Wang, Guo Xie

**Affiliations:** 1 School of Automation, Northwestern Polytechnical University, Xi'an, Shaanxi, China; 2 Department of Shenzhen Airport Terminal Area, Shenzhen Airport CO., Ltd., Shenzhen, Guangdong, China; South China University of Technology, CHINA

## Abstract

Disruptive behavior by passengers delayed at airport terminals not only affects personal safety but also reduces civil aviation efficiency and passenger satisfaction. This study investigated the causal mechanisms of disruptive behavior by delayed passengers in three aspects: environmental, managerial, and personal. Data on flight delays at Shenzhen Airport in 2018 were collected and analyzed. The main factors leading to disruptive behavior by delayed passengers were identified, and an early warning model for disturbances was developed using multiple logistic regression and a back-propagation(BP) neural network. The results indicated that the proposed model and method were feasible. Compared to the logistic regression model, the BP neural network model had advantages in predicting disturbances by delayed passengers, showing higher prediction accuracy. The BP network weight analysis method was used to obtain the influence weight of each factor on behavior change of delayed passengers. The influence weight of different factors was obtained, providing an assistant decision-making method to address disruption from flight-delayed passengers.

## 1 Introduction

China has become a prominent force in civil aviation, and since 2005 its air-transport volume has ranked second highest in the world [1]. However, along with such rapid development, China also increasingly faces the problem of flight delays. Flight delays, especially long ones, can lead to disruptive behavior by passengers. This behavior not only affects the normal operation of the airport but also threatens the safety and service quality of civil aviation. According to statistics from Shenzhen Airport Terminal Operation Center, factors such as weather and air traffic control problems cause thousands of delayed flights annually, leading to many incidents of disruptive behavior. Due to a lack of effective early warning methods, the civil aviation industry is often reactive in dealing with disruptive behavior. This not only wastes manpower and material resources but also has little effect on reducing incidences of disruptive behavior. Such behavior can lead to a loss of control at the airport and seriously affect civil aviation safety.

Disruptive passenger behavior is a complex issue. Liu suggested that an event involving groups of people involves complex social phenomena, and it is often the case that a key person initiates the event; thus, that person should be identified and premanaged to control the occurrence of group events [2]. Using qualitative methods, Dell’Olio et al. classified key variables found to be related to human group behavior in certain situations [3]. Studies of group events caused by flight delays have mainly used qualitative analysis. Such research has investigated the main factors in incidents through passenger satisfaction surveys and has proposed measures to reduce incidents from economic and legal perspectives [4, 5]. Thengvall et al. suggested that when flights are delayed, airlines and airports do not adequately respond to passengers’ demands, which increases anxiety and may lead to group events [6]. At present, research on the main factors affecting disruptive passenger behavior has rarely considered the characteristics of terminal management, and quantitative studies are few. As such, attempts to apply an early warning system to terminal management have been limited.

This study’s research team spent three months in a terminal management department to collect data on passenger disruption events related to flight delays. The factors causing passenger disruption were found to be varied, and the amount of each factor’s contribution was unclear. The relationships between factors could not be directly determined. In this regard, BP neural networks and multiple logistic regression have their own unique advantages for dealing with complex problems, and they have been widely used in research on industry, transportation, and medicine, among other fields [7–13]. Using a backpropagation (BP) neural network, Ahmed detected and classified faults in automobiles’ internal combustion engines and used experiments to verify the method’s stability and accuracy [14]. Wu used a BP neural network to analyze the measurement errors of an airborne laser and found that the method was beneficial for improving the accuracy of airborne ranging [15]. Zeng et al. developed a neural network (NN) model to explore the nonlinear relationship between crash frequency and risk factors[16]. Using four data-analysis methods, Reeve aimed to improve traditional logistic regression for analyzing treatment differences in incidence rates, thereby expanding upon logistic regression and generalizing the link function. Among the four methods, resampling based on the exact distribution function yielded the closest to nominal coverage rate [17].

Based on survey data, and drawing on prior experience providing on-site support in civil aviation, the present study analyzed the causes of disruptive behavior by delayed passengers to determine the key factors. Multiple logistic regression and a BP neural network were used to create a predictive model for disruptive passenger behavior; comparative experiments were then used to verify the model’s effectiveness.

## 2 Analysis of the main factors leading to disruptive behavior by delayed passengers

### 2.1 Causal mechanisms

Passenger behavior is an open, sudden, and complex system. When a complex system characterized by dynamic change is disturbed, it may change from a stable state to an unstable one. This process passes through a critical interface, which is the interface between two states. Between the stable state and the interface, the system is stable and controllable. Beyond the critical interface, the system is in an unstable state; it is then uncontrollable and cannot easily return to the original state [18].

When flights are delayed, passengers cannot travel normally, which results in psychological dissatisfaction. As passenger dissatisfaction becomes more serious, it reaches a critical state; once the critical state is exceeded, there is a risk of passenger disturbance. In this study, the critical factor refers to verbal disputes between passengers and staff in cases of flight delay. Disruptive passenger behavior is defined as extreme behavior that affects flight security or disrupts public order for the purpose of making demands or expressing dissatisfaction.

Many factors affect individual behavior in unconventional events, and the relationships among them are complex [19]. The causes of disruptive behavior are varied, and the circumstances and means of expression differ. This study collected flight delay data on-site from Shenzhen Airport in 2018, and passenger disturbance events were tracked and investigated in real time. Based on the obtained data, three main factors were identified: personal, environmental, and managerial.

#### (1) Personal

With improvements in living standards, people tend to prefer quicker and more convenient means of travel; thus, civil aviation has become a popular choice for long-distance travel. Due to intrinsic personal differences (e.g., occupation, age, physical health, mental health), passengers can react differently to flight delays. Thus, the likelihood of disruptive behavior can vary among different passengers.

#### (2) Environmental

*1) Time*. Passengers choose air travel mainly for convenience. Flight delays, however, offset the advantages and produce dissatisfaction. Generally, as the duration of flight delay increases, passenger dissatisfaction intensifies. Moreover, for physiological reasons, long-term delays at night can further increase dissatisfaction. Therefore, when analyzing the effect of time on passenger mood, we should consider both the duration and timing of the delay.

*2) Space*. Crowding in confined spaces can adversely affect people’s emotions and make them irrational. People are easily affected by the emotions of others around them, making it difficult to control their behaviors [20]. When flights are delayed, terminals may become crowded, and passenger disturbance incidents often occur in these crowded areas. Groups may form among passengers with similar demands. When a passenger begins to adopt language and actions reflecting the characteristics of a group leader, other passengers may unconsciously imitate the behavior. Under such a group dynamic, an individual who is usually calm and restrained may become disruptive.

#### (3) Managerial

When flights are delayed, service staff must deal with passengers directly. Different airlines provide different kinds of training and management for their employees, and the level of service can therefore vary. The occurrence of disruptive behavior is usually sudden, contingent, time sensitive, and wide ranging; thus, efficient management systems and sound plans are needed to deal with it in a timely and efficient manner. If staff have insufficient training and professional knowledge for dealing with emergencies, they will not effectively respond to passengers’ psychological changes, which could easily worsen passenger behaviors. Meanwhile, when a flight is delayed, the airline may provide some supplies to passengers. However, since the law does not specify compensation for delayed passengers, compensation can vary from airline to airline. Some airlines, for example, provide free food while others do not. These different levels of service can have different psychological effects on passengers.

**Fig 1:**
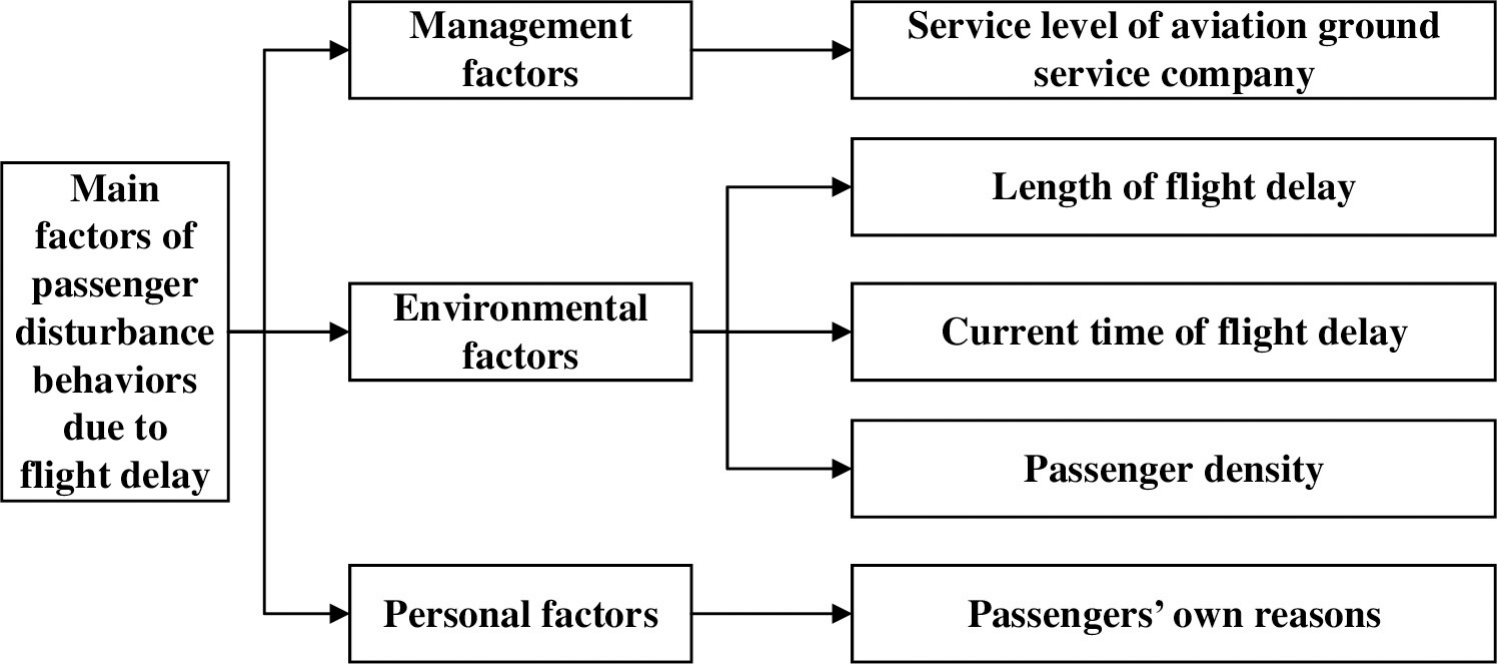
Main factors influencing disruptive behavior by passengers.

Fig 1 shows the main factors that lead to disruption by passengers in airport terminals.

### 2.2 Data sources and statistical analysis

The behavior states of delayed passengers in a terminal can be divided into three categories: emotional stability, quarrel, and disturbance. In the first state, passengers are very calm, waiting quietly without any disturbing behavior or verbal disputes. In the second state, there are verbal disputes between passengers and staff but no disturbances. In the third state, there are passenger disturbances.

The Civil Aviation Administration of China stipulates that a flight delay occurs when the actual departure time is at least 20 minutes later than the planned departure time. Accordingly, we collected data for 856 delayed flights from the terminal management department of Shenzhen Airport from June 1 to August 30, 2018. These included 355 flights without disruption, 391 flights with verbal disputes only, and 110 flights with disruptions. The specific effects of each category are analyzed below.

#### (1) Length of the flight delay

The 856 delayed flights, including 110 with passenger disruptions, were statistically analyzed based on the length of the delays (Table 1).

**Table 1:** Statistics on the length of flight delays and disruptive behaviors at Shenzhen Airport, 2018.

Flight delay time (h)	Number of delayed flights	Number of delayed flights with disturbances	Percentage (%)
**<1**	210	16	7.62
**1–2**	152	9	5.92
**2–3**	186	11	5.91
**3–4**	179	24	13.41
**>4**	129	50	38.76

#### (2) Time of flight delay

The likelihood of disturbance varies according to the time of day in which a delay occurs. Passengers become more anxious at night because they worry about whether they will arrive the same day and whether the airport can solve transportation and accommodation problems. The delayed flights, including 110 with passenger disruptions, were statistically analyzed based on the time of the flight delay (Table 2).

**Table 2:** Statistics on the time of flight delay and passenger disturbance at Shenzhen Airport, 2018.

Time of flight delay	Number of delayed flights	Number of delayed flights with disturbances	Percentage(%)
**6–10 a.m.**	259	6	2.43
**10 a.m.–2 p.m.**	280	19	6.73
**2–6 p.m.**	170	17	10.17
**6–10 p.m.**	123	27	21.72
**10 p.m.–2 a.m.**	110	36	32.86
**2–6 a.m.**	40	5	11.79

Since flight delay is a cumulative process, delayed flights in different time periods were partially repeated. Following this method, the number of delayed flights was more than 856.

#### (3) Passenger density at boarding gates

When there is a delay, flights can be allocated to different boarding gates. Each boarding gate has a different number of flights and a different passenger density, leading to differing likelihoods of disturbance. Table 3 shows the number of delayed flights allocated to boarding gates and the number of disturbances at Shenzhen Airport in 2018. The statistical analysis indicated that the more flights allocated to the same gate, the higher the passenger density, and the greater the likelihood of disturbance incidents.

**Table 3:** Statistics on the number of delayed flights allocated to boarding gates and passenger disturbances at Shenzhen Airport, 2018.

Number of flights allocated to a boarding gate	Number of delayed flights	Number of delayed flights with disturbances	Percentage(%)
**1–2**	398	20	4.97
**3–4**	344	54	15.70
**≥5**	114	36	31.58

#### (4) Service level of aviation ground service companies

Airline passenger satisfaction scores for 2018 were collected from the website of the Civil Aviation Passenger Service Satisfaction Survey (https://www.capse.net/). The data were classified and counted, and Table 4 shows the statistical results.

**Table 4:** Delayed flights and satisfaction evaluation scores of different airlines at Shenzhen Airport, 2018.

Satisfaction score	Number of delayed flights	Number of delayed flights with disturbances	Percentage(%)
**<3.5**	279	62	22.22
**3.5–4**	267	34	12.73
**>4**	310	14	4.52

#### (5) Average age of delayed flight passengers

The average age of delayed flight passengers was 30–45. Table 5 shows the data for the average age of delayed passengers and the number of delayed flights with disturbances.

**Table 5:** Average age of delayed flight passengers and the number of flights with passenger disruptions at Shenzhen Airport, 2018.

Average age of delayed flight passengers	Number of delayed flights	Number of delayed flights with disturbances	Percentage(%)
**<35**	113	25	22.12
**35–40**	326	40	12.27
**>40**	417	45	10.79

## 3 Prediction model based on multiple logistic regression

### 3.1 Multiple logistic regression

Logistic regression is a kind of linear regression. The basic principle is to use the function *f*(*z*) as the function to predict by linearly summing the prediction factors. The value of *f*(*z*) is [0, 1]. When the event occurs, probability is *p*; when the event does not occur, probability is 1-*p*. Maximum likelihood estimation is used for parameter calculation. Multiple logistic regression is a multivariate statistical analysis method used to analyze the relationship between dependent (reaction) and independent (observation) variables in multiclassification situations [21]. The number of independent variables is *n*, and the number of dependent variables is *m*, where *m*≥3. The principle of multiple logistic regression is to divide different factor classifications into multiple binary logistic regressions. Its mathematical model is described as follows:

$$f(z_{j})=e^{z_{j}}/(1+e^{z_{j}}),$$ (1)

$$z_{j}=\alpha_{j}+\sum_{k=1}^{n}\beta_{jk}x_{k},$$ (2)

$$P(y=j|x)=\frac{e^{\alpha_{j}+\sum_{k=1}^{n}\beta_{jk}x_{k}}}{1+\sum_{j=1}^{m-1}e^{\alpha_{j}+\sum_{k=1}^{n}\beta_{jk}x_{k}}},$$ (3)

$$\ln\left[\frac{P(y=j|x)}{1-P(y=j|x)}\right]=\alpha_{j}+\sum_{k=1}^{n}\beta_{jk}x_{k},$$ (4)

$$P(y=j|x)=\pi_{j},$$ (5)

$$\sum_{j=1}^{m}\pi_{j}=1,$$ (6)

where, *P* is the probability of the occurrence of reaction variable type *j*, *x*_*k*_ is observation variable *k*, *β*_*jk*_ is the regression coefficient, *α*_*j*_ is the regression intercept of reaction variable *j*, and *π*_*j*_ is the conditional probability of the occurrence of event *j*.

### 3.2 Establishment of delayed flight dataset

Through the analysis set forth in Section 2.2, we obtained five factors that affect the incidence of passenger disturbance. Based on these factors, we can establish the following delayed flight data set for analysis:

$$X_{i}=\{x_{i}(1),x_{i}(2),x_{i}(3),x_{i}(4),x_{i}(5)\},$$ (7)

where *X*_*i*_ is the vector of data for delayed flight *i*, *x*_*i*_(1) is length of the delay for flight *i*, *x*_*i*_(2) is time of flight delay *i*, *x*_*i*_(3) is passenger density at the boarding gate for delayed flight *i*, *x*_*i*_(4) is service level of aviation ground service companies for delayed flight *i*, and *x*_*i*_(5) is average age of delayed flight passengers for flight *i*.

### 3.3 Experimental analysis

Passenger behavior state is the dependent variable, and the influencing factor is the independent variable. The confidence interval of the model is 95%. Multiple logistic regression analysis was carried out using SPSS analysis tool.

The significance level of each factor and model was less than 0.05. The model had statistical significance, and the selected factors were effective (Tables 6 and 7). The overall prediction accuracy of the model was 71.03%, as shown in Table 8.

**Table 6:** Model fitting information.

Model	Model fitting standard	Likelihood ratio test		
	−2 Log Likelihood	Chi-square	df	Sig.
**Intercept only**	1389.254			
**Final**	922.427	466.827	30	0.000

**Table 7:** Maximum likelihood ratio test of parameters.

Parameters	−2 Log Likelihood	Chi-square	df	Sig.
**Intercept**	922.427	0.000	0	0.000.
**Length of flight delay**	1152.750	230.325	8	0.000
**Time of flight delay**	983.544	61.117	10	0.000
**Passenger density at boarding gates**	1009.550	87.123	4	0.000
**Average age of delayed flight passengers**	943.329	11.903	4	0.018
**Service level of aviation ground service companies**	948.969	26.542	4	0.000

**Table 8:** Model prediction accuracy.

Observed value	Measured value			
	Disturbance	Quarrel	Emotional stability	Percentage (%)
**Disturbance**	15	75	20	13.64
**Quarrel**	12	325	54	83.12
**Emotional stability**	2	85	268	75.49
**Prediction accuracy**				71.03

## 4 Prediction model based on BP neural network

### 4.1 BP neural network

Section 3 analyzes the factors related to disruptive delayed-passenger behavior, finding complex interaction between the factors and disturbance-incident occurrence. This section establishes a disturbance early warning model.

**Fig 2:**
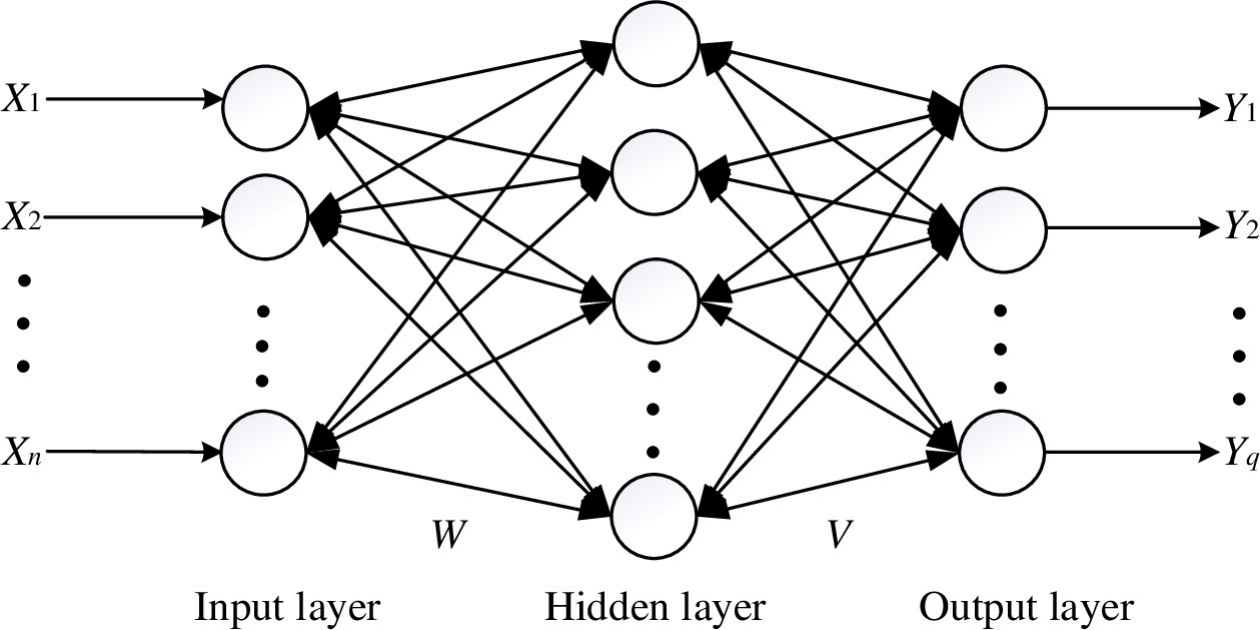
Logical structure of an early warning model algorithm based on a single hidden-layer BP neural network.

A BP neural network consists of a multilayer feed-forward neural network based on an error backpropagation algorithm. It was pioneered by Rumelhart and McClelland in 1986 [22] and has become the most widely used neural network learning algorithm [23–26]. The BP neural network consists of one input layer, one or more hidden layers, and one output layer. Each layer has one or more neurons. Information is transmitted from the input layer to the output layer through the hidden layer or layers. The strength of the connection between neurons in different layers is represented by a connection weight [27]. Taking a BP neural network with a single hidden layer as an example, its logical structure is shown in Fig 2.

The principle of the BP neural network is as follows:

$$X^{k}={\left(X_{1}{}^{k},\;X_{2}{}^{k},\;\cdots ,\;X_{n}{}^{k}\right)}^{T}(k=1,2,\cdots ,m)$$ (8)

$$Y^{k}={\left(Y_{1}{}^{k},\;Y_{2}{}^{k},\;\cdots ,\;Y_{q}{}^{k}\right)}^{T}$$ (9)

$$S^{k}={\left(S_{1}{}^{k},\;S_{2}{}^{k},\;\cdots ,\;S_{p}{}^{k}\right)}^{T}$$ (10)

$$B^{k}={\left(B_{1}{}^{k},\;B_{2}{}^{k},\;\cdots ,\;B_{p}{}^{k}\right)}^{T}$$ (11)

$$L^{k}={\left(L_{1}{}^{k},\;L_{2}{}^{k},\;\cdots ,\;L_{q}{}^{k}\right)}^{T}$$ (12)

$$C^{k}={\left(C_{1}{}^{k},\;C_{2}{}^{k},\;\cdots ,\;C_{q}{}^{k}\right)}^{T}$$ (13)

$$W=\{W_{ij}\}(i=1,2,\cdots ,n;j=1,2,\cdots ,p)$$ (14)

$$V=\{V_{jt}\}(j=1,2,.\cdots ,p;t=1,2,.\cdots ,q),$$ (15)

where *X*^*k*^ is the input vector, *Y*^*k*^ is the output vector, *m* is the number of learning mode pairs, *n* is the number of units of the input layer, *q* is the number of units of the output layer, *p* is the number of units of the hidden layer, *S*^*k*^ is the net input vector of the hidden layer, *B*^*k*^ is the net output vector of the hidden layer, *L*^*k*^ is the net input vector of the output layer, *C*^*k*^ is the actual output vector of the output layer, *W* is the connection weights of the input and hidden layers, and *V* is the connection weights of the hidden and output layers.

### 4.2 Designing the BP neural network

Based on the analysis in Section 3.2, a quantization process was performed to obtain a matrix of impact factors. As a result, there are five nodes on the input layer and one node on the output layer. Since a single hidden-layer neural network can theoretically approximate any continuous function (as long as there are a sufficient number of neurons in the hidden layer) [28], this study used a single hidden layer to construct the early warning model.

#### (1) Design of the hidden layer

Determining the number of neurons in the hidden layer is very important in the network design process. Too many neurons will increase the amount of processing and lead to over fitting. If the number of neurons is too small, it will affect network performance and may not achieve the expected results. The number of hidden-layer neurons is related to the complexity of the problem, the number of neurons in the input and output layers, and the setting of the expected error. In this study, an empirical formula was used as a guideline for the initial selection of the number *l* of neurons in the hidden layer [29]:

$$l=\sqrt{n+m}+a$$ (16)

a∈[1,10], (17)

where *n* is the number of neurons in the input layer, *m* is the number of neurons in the output layer, and *a* is a constant adjusted based on the accuracy and convergence speed of the verification stage.

#### (2) Selection of activation function

A BP neural network often uses sigmoid-type differentiable functions or linear functions as the activation functions for the network. TANSIG and LOGSIG are types of sigmoid functions. This study chose the TANSIG function as the transfer function for neurons in the hidden layer. Since the output of the network was normalized to the range [−1, 1], the prediction model also applied an S-shaped logarithm function TANSIG as the transfer function for neurons in the output layer.

### 4.3 Weight analysis of influencing factors

The weight analysis of influencing factors refers to calculating the proportion of the connection weights of input nodes related to input factors in the total weights of all input nodes to the output contribution of the network. According to the weight contribution rate, the degree of influence of the input factors on the output is judged, so as to determine its importance. A single hidden layer BP neural network with 5 input nodes and 1 output node is constructed. The weight calculation formula [30] is as follows:

$$b_{i}=\sum_{j}|W_{ij}|\cdot [|V_{j}|\cdot (\ln|W_{ij}|/\ln\sum_{i}\left|W_{ij}\right|)]$$ (18)

$$c_{i}=b_{i}/\sum_{i}b_{i},$$ (19)

where *b*_*i*_ is the weight contribution rate of input node *i*, *W*_*ij*_ is the connection weights of input layer node *i* and hidden layer node *j*, *V*_*j*_ is the connection weight between hidden layer node *j* and output node *q* (*q* = 1), and *c*_*i*_ is the weight contribution rate of input node *i*.

### 4.4 Data standardization

Input data standardization was conducted on the basis of Sections 2.2 and 3.2 (Table 9). Whether disruptive behavior occurred was the target for the output prediction of the BP neural network. This is represented by the variable *Y* with the range [0.25, 0.5, 0.75]. An output of 0.25 indicates emotional stability, 0.5 quarrel, and 0.75 disturbance.

**Table 9:** Data standardization of BP neural network input layer.

Input variable		Standardized value
**Length of flight delays (*X***_**1**_**)**	<1	0.2
	1–2	0.4
	2–3	0.6
	3–4	0.8
	>4	1
**Time of flight delays (X**_**2**_**)**	2–6	0.4
	6–10	0.1
	10–14	0.2
	14–18	0.3
	18–22	0.7
	22–02	1
**Average age of delayed flight passengers (X**_**3**_**)**	<35	0.3
	35–40	0.6
	>40	0.9
**Passenger density at boarding gates (*X***_**4**_**)**	1–2	0.1
	3–4	0.5
	≥5	1
**Service level of aviation ground service companies (*X***_**5**_**)**	<3.5	1
	3.5–4	0.6
	>4	0.2

### 4.5 Results

In accordance with Section 2.2, data were collected for 856 delayed flights. Data for 227 flights were randomly selected as test data. Target error was set to 0.01, learning rate to 0.02, and training accuracy to 0.001. Error, defined as the absolute value of the difference between test output *Y*’ and expected value *Y*, should not be greater than 0.125. If this value is greater than 0.125, the prediction is counted as an error.

In fact, if there are noise data in the input neural network data, too many neurons will make the noise data influence amplification, leading to reduced prediction accuracy of the model and the over-fitting phenomenon. The results showed that for the single hidden-layer BP network, the accuracy of the trained model increased with the number of neurons and the number of training iterations. However, when the increase reached a certain level, the rate of change leveled off. Table 10 shows the results.

**Table 10:** BP neural network prediction errors.

Number of neurons	Number of learning cycles	Number of errors exceeding 0.125	Percentage error (%)	Prediction accuracy (%)
7	10000	77	33.92	66.08
	50000	67	29.52	70.48
	100000	62	27.31	72.69
	120000	61	26.87	73.13
13	10000	63	27.75	72.25
	50000	60	26.43	73.57
	100000	59	25.99	74.01
	120000	58	25.55	74.45
18	10000	61	26.87	73.13
	50000	56	24.67	75.33
	100000	50	22.03	77.97
	120000	47	20.70	79.30
20	10000	62	27.31	72.69
	50000	48	21.15	78.85
	100000	47	20.70	79.30
	120000	42	18.50	81.50
23	10000	56	24.67	75.33
	50000	47	20.70	79.30
	100000	49	21.59	78.41
	120000	48	21.15	78.85

**Fig 3:**
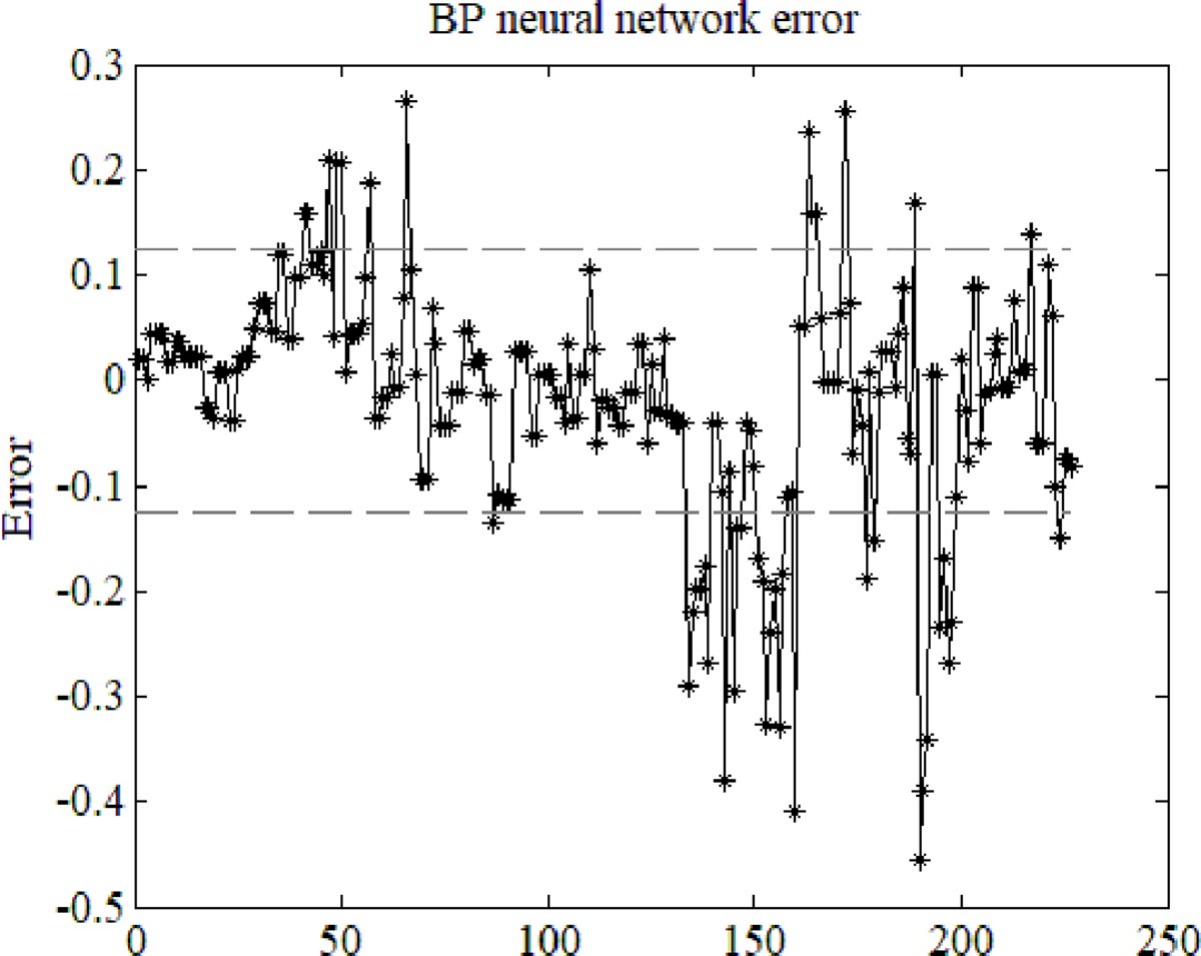
Prediction error with 20 neurons and 120,000 training iterations.

When the number of neurons was 7, and the number of training cycles was 10,000, the number of errors was 77 (33.92%). When the number of neurons was 13, and the number of training cycles was 100,000, errors were reduced to 25.99%, and accuracy reached 74.01%. When the number of neurons was 20, and the number of training cycles was 120,000, errors were reduced to 18.50%, and accuracy reached 81.50%. Fig 3 shows the corresponding actual network error at this time.

In accordance with Section 4.3, the influence weight of each factor is shown in Table 11. When the number of nerves was 20 and the number of training cycles was 120,000, influence weight of length of flight delays was 0.26, the influence weight of passenger density at boarding gates was 0.25, and the influence weight of average age of delayed flight passengers was 0.12, the smallest influence weight.

**Table 11:** Weight of factors for flight delay passenger behavior status.

Factors	Weight
**Length of flight delays**	0.26
**Time of flight delays**	0.19
**Average age of delayed flight passengers**	0.12
**Passenger density at boarding gates**	0.25
**Service level of aviation ground service companies**	0.18

## 5 Conclusion

Combining on-site data and a situational assessment of civil aviation, this study analyzed the causal mechanisms of delayed-passenger disruptions in terms of personal, environmental, and managerial aspects. Based on 2018 data for flight delays at Shenzhen Airport, the main factors leading to delayed-passenger disruption were identified as follows: length of flight delay, time of flight delay, passenger density at boarding gates, service level of aviation ground service companies, and intrinsic passenger factors. A BP neural network and multiple logistic regression were used to establish prediction models. The experimental results indicated that the prediction accuracy of the BP neural network reached 81.5%, showing a better prediction effect than multiple logistic models. According to the influence weight analysis of the BP neural network, the length of flight delays and passenger density at boarding gates have a great impact on the behavior of delayed passengers. In practice, airport staff should pay more attention to passengers experiencing long delays. When flight density at the gate is too high, measures such as increasing service personnel should be taken to avoid passenger disturbance behavior.

As shown in the statistics, disturbances caused by passenger’s own reasons were difficult to quantify, only the average age of passengers was quantified. In the early warning analysis of passenger disturbances, priority was given to factors that had a greater impact on actual operating processes. Meanwhile, factors that were more difficult to observe—such as the physical and mental states of passengers—were not considered. In the future, more individual passenger factors should be integrated into the research, and the training parameters should be further optimized. Thus, there is a need for further research on the selection of impact factors. In addition, more methods will be used to establish the prediction model, such as ordered logistic regression, which is a direction of the future research. The prediction accuracy will be compared with that of BP neural network.
